# Characteristics and Risk Factors for Spontaneous Closure of Idiopathic Full-Thickness Macular Hole

**DOI:** 10.1155/2019/4793764

**Published:** 2019-03-13

**Authors:** Xida Liang, Wu Liu

**Affiliations:** Beijing Tongren Eye Center, Beijing Tongren Hospital, Beijing Ophthalmology and Visual Sciences Key Laboratory, Capital Medical University, Beijing 100730, China

## Abstract

Idiopathic full-thickness macular hole is a severe visual impairment disease. Pars plana vitrectomy remains the primary treatment option for idiopathic full-thickness macular holes, and over 90% idiopathic full-thickness macular holes are closed by vitrectomy surgery. However, the full-thickness macular hole may close spontaneously, with a good visual acuity progress. Since recent studies are small-sample studies and case reports, the characteristics for spontaneous closure of idiopathic full-thickness macular holes are not fully understood. Here, we review the articles in PubMed database from 1999 to 2018 and discuss the characteristic and the risk factors, especially OCT structure features, for spontaneous closure of idiopathic full-thickness macular holes.

## 1. Introduction

Idiopathic macular hole (IMH) is a kind of disease that seriously affects the visual quality of patients, showing obvious central visual loss and visual deformation. The AAO guidelines suggest that stage 2–4 IMHs have a good prognosis after vitrectomy surgery, which can effectively improve the central vision acuity and reduce metamorphopsia. The closure rate after vitrectomy has been reported to be from 91% to 98% in the last 5 years [1–4]. Currently, vitrectomy is recognized as a primary and effective treatment option. However, it is noted that the IMH can spontaneously close without any intervention, which has been consistently reported in recent years. The mechanism of spontaneous closure of IMHs and the risk factors have not been fully elucidated. Therefore, it is necessary to summarize the general characteristics and OCT structural features of spontaneous closure of IMHs and discuss the risk factors of spontaneous closure of full-thickness IMHs.

A structured literature search in PubMed was performed on October 1, 2018, and a search strategy of “((spontaneous closure) OR (spontaneous closed) OR (spontaneous resolution)) AND (macular hole)” executed in “title and/or abstract” was applied. We also checked all the references of relevant reviews and eligible articles that our search retrieved. Language restrictions were not used. Title and abstract screening of all retrieved articles was performed using prespecified criteria. Studies were included if all following criteria were fulfilled: (1) adults with IMHs and (2) the IMH closed spontaneously during the follow-ups. Criteria for exclusion were as follows: (1) adults with systemic diseases, such as diabetes and hypertension; (2) adults with ophthalmic diseases, glaucoma, high myopia, diabetic retinopathy, etc.; (3) adults with a history of ophthalmology surgery; and (4) surgery and drug intervention performed during the period from the onset of IMHs to closure.

We identified and screened a total of 186 articles and found 78 relevant articles. Among these, 30 articles were excluded for non-IMHs. 11 articles were excluded because patients also suffered from other ophthalmic diseases. 8 articles were excluded because the cases were not described in detail. Finally, we included 21 articles, including 58 eyes in 54 patients.

In our statistical result, the average age of spontaneous closure of IMHs was 69.0 ± 6.4 (50–81). Complete data on gender were recorded in 38 reports, with 26 females and 12 males. The stages of IMHs were described in 41 eyes, with 21 stage II, 13 stage III, and 7 stage IV IMHs. The course of disease (the time between patients' description of symptoms and visit time) ranged from 5 days to 31 months, with an average of 96.1 ± 178.0 d. The closure time was 100.9 ± 98.3 d on average. Follow-up time ranged from 3 months to 49 months. The average initial BCVA was 0.36 ± 0.20, and the average final BCVA after spontaneous closure of MH was 0.70 ± 0.17. The average diameter of MH was 178.6 ± 77.3 *μ*m. The results of this query are shown in Table 1.

## 2. Incidence

The incidence of spontaneous closure of idiopathic macular holes varies from 4% to 11.5%. Yuzawa et al. retrospected 97 eyes with full-thickness IMH for a period ranging from 2 to 182 months, and 6 eyes (6.2%) showed spontaneous closure within 24 months of the initial examination [26]. A multicentered, controlled, randomized clinical trial by Freeman et al. studied 129 eyes of 122 patients with stage III or IV IMH [27]. In their studies, 64 eyes were randomized to surgery, and 65 eyes were randomized to observation. 2 (4%) of the holes in 56 eyes randomized to observation were spontaneously closed. Another randomized clinical trial by Ezra et al. studied 185 eyes of 174 patients, which also randomized to surgery group and observation group [28]. Their results showed that spontaneous closure of the FTMH occurred in 7 (11.5%) of 61 patients, with little or no change in overall acuity levels in 24 months. In 2012, Okubo et al. retrospectively reviewed the records of 142 eyes of 138 patients with full-thickness IMH [8]. The full-thickness IMHs were diagnosed by OCT with 45 stage II eyes (31.7%), 71 stage III eyes (50%), and 26 stage IV eyes (18.3%) using OCT. They found five eyes (3.5%) with spontaneous closure. However, vitrectomy was planned in all eyes between 11 days and 154 days (mean: 65.2 days) after the initial presentation, which means part of the IMHs may not have enough observation time. In conclusion, the incidence of spontaneous closure ranges from 4% to 11.5%, and further studies for a relatively fixation observation time by OCT will be needed to verify the incidence results.

## 3. Age, Gender, and Closure Time

So far, no researches have mentioned that basal characteristics, such as age, gender, and the course of disease, have relationship with spontaneous closure of IMHs. Among these, the age of patients reported by most of the articles was greater than 60, except a 50-year-old case reported by Kelkar et al. [9]. The average spontaneous closure time of IMHs was 99.5 ± 98.0 d. Morawski et al. [7] suggested a 2- to 3-month observation time before surgery in posttraumatic eyes, since a quite number of cases showed spontaneous closure. In our review data, it seems that a 3-month observation time may also be considered in IMHs. However, it should be mentioned that the course of disease (the time between patients' description of symptoms and visit time) varies from 5 days to 31 months, which create biases in suggesting observation time.

## 4. Visual Acuity

All cases with a spontaneous closure of IMHs acquired progress in visual acuity. The average initial BCVA was 0.36 ± 0.20, and the average final BCVA after spontaneous closure of MH was 0.70 ± 0.17. This shows that the spontaneous closure of MH may gain a well visual acuity, and the influencing characteristic needs to be explored.

## 5. IMH Diameter

Many articles mentioned that the spontaneous closure occurred in IMHs with a relatively small diameter. Sugiyama et al. suggested that MHs of less than 250 *μ*m diameter have more opportunity to close spontaneously [11]. Privat et al. believed that the diameter of MHs is probably the main factor for the spontaneous closure, since the diameters in their study are between 70 and 250 *μ*m in 13 patients, except in one patient who had a 350 *μ*m macular hole [21]. In our review data, the diameter of 34 IMHs are smaller than 250 *μ*m and that in 13 IMHs are between 250 *μ*m and 400 *μ*m. It seems that the small diameter of IMHs gives the chance to both edges of the IMHs to combine together. Also, in a case studied by Fernández and Navarro [10], the minimum diameter of IMHs is much smaller than basal diameter. They suggested that this may be a recovery phenomenon, which indicates the inner layer of macular retinal concentrates to the center. Although most studies did not provide metrical data of basal diameter, we observed that about half of the IMHs shows much larger basal diameter than minimum diameter. However, the hypothesis needs to be confirmed by a larger sample study.

## 6. VMT

It is widely believed that spontaneous release of the vitreomacular traction (VMT) may account for the closure of IMHs [21], which is also the theory of vitrectomy surgery, since many MHs closed after the relieve of VMT [5, 6, 8–10]. Indeed, in our review data, more than half of the IMHs were in stage II, and most VMT relieve during their spontaneous closure of IMHs. However, Privat et al. also mentioned 6 stage III or IV MHs in which vitreous already detached from the MH edge at the first examination [21]. In Morawski et al.'s study, 5 out of 9 eyes (one excluded for trauma history) showed no vitreous detachment in foveal during closure [7]. Since the VMT was relieved in stage III and stage IV IMHs, the mechanism of spontaneous closure may be complicated. Fernández and Navarro thought this phenomenon may due to the different first examine time, which means these macular holes may already have undergone spontaneous closing after the relief of VMT [10]. Kelkar et al. reported a stage II full-thickness MH spontaneously closed without the relieve of VMT, which is also shown in the report of Freund et al. [9, 29]. These results indicate that the relieve of VMT promotes the spontaneous closure in IMHs, but it may not be the indispensable reason.

## 7. Sharp Edge and Bridge-Like Structure

Previous literature has suggested that some of the edges of macular holes become sharp and stretch out bridge-like structure. This sharp edge was stated by Michalewska et al. [17], who believed the phenomenon may facilitate the spontaneous closure. Morawski et al. [7] supposed that the sharp edge facilitates bridging and spontaneous MH closure. We statistically analyzed the results of OCT images in the cases and found that 27 cases (69%) had obvious sharp edges, while 12 cases did not. It has been reported that this may be the result of collagen secretion by outer retinal cells such as Müller cells. The extension and proliferation of the Müller cells may also form this bridge-like structure [8]. We speculate that the occurrence of this phenomenon may be gradually obvious with the occurrence of spontaneous closure. Due to the differences in the follow-up time between the cases, the 12 cases without obvious bridge-like structure may miss the phenomenon occurring time. Therefore, the bridge-like structure may be an important manifestation of spontaneous closure process. However, we reviewed some cases of macular hole occurrence that were not closed, and the appearance of this bridge-like structure was also observed. Therefore, the bridge-like structure may be an important, but maybe not unique, manifestation during the spontaneous closure process of macular holes.

## 8. Epiretinal Membrane Formation

Few articles found the epiretinal membrane formation during the spontaneous closure of MH. Smiddy [30] and Petropoulos et al. [15] each found one IMH with epiretinal membrane formed during the spontaneous closing process, but this phenomenon has not been found in other cases at present. Petropoulos et al. believed that the formation of a contractile epiretinal membrane can facilitate the macular hole closure. However, the epiretinal membrane formation was not widely seen in spontaneously closed macular holes. This suggests that epiretinal membrane formation may be one of the reasons for spontaneous closure of IMHs.

## 9. Cystic Structure

In the meantime, there are some spontaneously closed macular holes with cystic structure [17]. This kind of macular hole has a thicker fovea, and the thickness of the macular hole decreases obviously after the spontaneous closure. RPE cells have the function of absorbing extracellular fluid in the cyst cavity [17]. We speculate that the closure of this kind of macular hole may be related to the absorption of cystic structure.

## 10. Autofluorescence and OCT Angiography

Resent studies has focus on the autofluorescence and OCT angiography in macular hole closure. Zhang et al. believed that postoperative area of high AF in macula can be an evaluating indicator for poor macular function recovery [31]. Teng et al. suggested that choroidal circulation in the macular area, which is detected by OCT angiography, might be affected by the intact structure of the fovea [32]. However, whether these characteristics have correlations with spontaneous closure of macular holes remains unclear.

## 11. Conclusion

In conclusion, there exists a percentage of idiopathic full-thickness macular holes to be spontaneously closed, which occur in about 3 to 4 months after initial examination. The macular hole with the diameter of less than 400 *μ*m, especially less than 250 *μ*m, may have more chance to close spontaneously. Also, these spontaneously closed macular holes have some distinctive OCT characteristics, such as the relieve of VMT, bridge-like structure, epiretinal membrane, and cystic structure. In our review results, these characteristics give the macular hole tendency to close spontaneously. Although these characteristics may not be irreplaceable and unique risk factors, they give the doctor a consideration to observe for a few months before surgery. Further research is needed to understand the pathophysiology underlying the development of spontaneous closure of idiopathic full-thickness macular holes [33, 34].

## Figures and Tables

**Table 1:** Literature review of articles on spontaneous closure of idiopathic full-thickness macular holes.

No.	Author	Year	Age	Gender	Stage	Diameter	VMT	Bridge	CD time	SC time	Follow-up	EZ recover time	Initial BCVA	Final BCVA
1OD	Gonzalez-Cortes et al. [5]	2018	67	F	4	136	y	n	3 w	4 w	12 m	4 w	20/40	20/20
1OS	Gonzalez-Cortes et al. [5]	2018	67	F	2	272	n	y	3 w	4 w	12 m	20 w	20/60	20/25
2	Zvornicanin, et al. [6]	2017	64	M		213	y	y	5 d	56 d	6 m	56 d	20/80	20/20
3a	Morawski et al. [7]	2016	64			<250	n	y	28 w	32 w			20/40	20/50
3b	Morawski et al. [7]	2016	66			250–400	y	y	10 w	8 w			20/150	20/25
3c	Morawski et al. [7]	2016	74			<250	y	y	20 w	64 w			20/50	20/30
3d	Morawski et al. [7]	2016	65			<250	n	y	12 w	12 w			20/60	20/80
3e	Morawski et al. [7]	2016	81			<250	n	y	6 w	40 w			20/100	20/25
3f	Morawski et al. [7]	2016	66			<250	n	y	8 w	12 w			20/40	20/30
3g	Morawski et al. [7]	2016	70			<250	y	y	2 w	8 w			20/100	20/40
3h	Morawski et al. [7]	2016	75			250–400	y	y	24 w	48 w			20/80	20/50
3i	Morawski et al. [7]	2016	79			250–400	n	y	8 w	8 w			20/100	20/100
4	Okubo et al. [8]	2013	71	M	2	396 (b)	y	y	5 w	5 m	7 m	—	20/80	20/40
5	Kelkar et al. [9]	2013	50	F	2		n	n	1 m	6 w	3 m	—	20/125	20/30
6	Fernández and Navarro [10]	2012	67	M	4	60	n	y	11 m	3 m	2 y	7 m	0.5	0.7
7a	Sugiyama et al. [11]	2012	64	F	3	150				57 d	85 d		0.8	0.8
7b	Sugiyama et al. [11]	2012	62	M	2	240				49 d	226 d		0.2	0.6
7c	Sugiyama et al. [11]	2012	71	M	4	125				70 d	511 d		0.4	0.8
7dOD	Sugiyama et al. [11]	2012	72	M	2	250				56 d	1146 d		0.1	0.8
7dOS	Sugiyama et al. [11]	2012	72	M	3	210				70 d	579 d		0.4	0.9
8a	Inoue et al. [12]	2012	78	F	4	135		n		3 m	40 m	6–9 m	20/40	20/20
8b	Inoue et al. [12]	2012	76	F	2	280		n		2 m	36 m	9–12 m	20/100	20/25
8c	Inoue et al. [12]	2012	73	M	2	156		y		3 m	49 m	3–9 m	20/50	20/20
8d	Inoue et al. [12]	2012	65	F	2	333		y		1 m	43 m	33–36 m	20/63	20/32
8e	Inoue et al. [12]	2012	73	M	3	197		n		3 m	42 m	6–9 m	20/100	20/30
8f	Inoue et al. [12]	2012	65	F	3	152		n		4 m	36 m	1–4 m	20/100	20/30
9	Lipkova et al. [13]	2011	65	F					31 m				20/100	20/60
10OS	Imasawa et al. [14]	2010	72	M	2	210	y	y	10 d	2 m	37 m	6 m	0.4	0.9
10OD	Imasawa et al. [14]	2012	72	M	3	250		n		2 m	19 m	6 m	0.1	0.7
11	Petropoulos et al. [15]	2009	63	M				n		2 m	12 m	5 m	0.6	1
12	Chen et al. [16]	2008	71	M	3	524 (b)	n	n	3 m	9 m	27 m	27 m	20/50	20/15
13a	Michalewska et al. [17]	2008	67	F	4	289	n	y	3 w	3 m	3 m		0.05	0.5
13bOD	Michalewska et al. [17]	2008	72	M	3	87	n	y		1 m	2 m	2 m	0.5	0.8
13bOS	Michalewska et al. [17]	2008				274	n	n		1 m	2 m		0.2	0.5
14	Milani et al. [18]	2007	70	F	2	50–150	y	y	2 m	10 m	15 m	10 m	0.3	0.8
15a	Hamano et al. [19]	2007	63	F	2	95		y		6 w	5 m	5 m	20/40	20/20
15b	Hamano et al. [19]	2007	59	F	4	70		y		3 w	8 w	8 w	20/100	20/30
16	Schweitzer and García [20]	2007	76	F	3	200	n	n	2 w	1 m	9 m	1 m	20/60	20/25
17a	Privat et al. [21]	2007	72	M	2	200				14 m			0.05	0.2
17b	Privat et al. [21]	2007	79	F	3	350				2 m			0.4	0.7
17c	Privat et al. [21]	2007	77	M	3	95				4 m			0.6	0.9
17d	Privat et al. [21]	2007	74	F	2	250				1 m			0.2	0.6
17e	Privat et al. [21]	2007	60	F	3	160				3 m			0.4	0.9
17f	Privat et al. [21]	2007	73	F	2	200		y		3 m			0.8	0.8
17g	Privat et al. [21]	2007	70	M	2	200		y		2 m			0.3	0.6
17h	Privat et al. [21]	2007	65	F	2	90				3 m			0.4	0.5
17i	Privat et al. [21]	2007	81	M	3	80				1 m			0.5	0.6
17j	Privat et al. [21]	2007	71	F	2	100				3 m			0.3	0.8
17k	Privat et al. [21]	2007	61	M	2	120		y		1 m			0.3	0.6
17l	Privat et al. [21]	2007	75	M	4	70				7 m			0.5	0.6
17m	Privat et al. [21]	2007	70	F	3	250				1 m			0.3	0.5
17n	Privat et al. [21]	2007	62	F	2	100				2 m			0.2	0.8
18a	Punjabi et al. [22]	2007	66	F	2			y	7 d	3 m	3 m	3 m	20/40	20/25
18b	Punjabi et al. [22]	2007	80	M	2			n	30 d	4 m	4 m	—	20/50	20/25
19	Win and Young [23]	2007	65	F				y	2 m	1 m	1 m		20/70	20/30
20	Lai et al. [24]	2006	62	F		137		y	2 w	3 w	3 w		20/40	20/30
21a	Ishida et al. [25]	2004	62	F					1 m	5 w	7 m	5 m	20/67	20/20
21b	Ishida et al. [25]	2004	65	F					2 m	3 m	3 m		20/33	20/22
